# The value of including boys in an HPV vaccination programme: a cost-effectiveness analysis in a low-resource setting

**DOI:** 10.1038/sj.bjc.6604023

**Published:** 2007-10-09

**Authors:** J J Kim, B Andres-Beck, S J Goldie

**Affiliations:** 1 Department of Health Policy and Management, Harvard School of Public Health, Program in Health Decision Science, 718 Huntington Avenue, 2nd Floor, Boston, MA 02115, USA

**Keywords:** cost-effectiveness analysis, HPV vaccination, cervical cancer, mathematical models, dynamic models, computer simulation

## Abstract

We assessed the cost-effectiveness of including boys *vs* girls alone in a pre-adolescent vaccination programme against human papillomavirus (HPV) types 16 and 18 in Brazil. Using demographic, epidemiological, and cancer data from Brazil, we developed a dynamic transmission model of HPV infection between males and females. Model-projected reductions in HPV incidence under different vaccination scenarios were applied to a stochastic model of cervical carcinogenesis to project lifetime costs and benefits. We assumed vaccination prevented HPV-16 and -18 infections in individuals not previously infected, and protection was lifelong. Coverage was varied from 0-90% in both genders, and cost per-vaccinated individual was varied from I$25 to 400. At 90% coverage, vaccinating girls alone reduced cancer risk by 63%; including boys at this coverage level provided only 4% further cancer reduction. At a cost per-vaccinated individual of $50, vaccinating girls alone was <$200 per year of life saved (YLS), while including boys ranged from $810–18 650 per YLS depending on coverage. For all coverage levels, increasing coverage in girls was more effective and less costly than including boys in the vaccination programme. In a resource-constrained setting such as Brazil, our results support that the first priority in reducing cervical cancer mortality should be to vaccinate pre-adolescent girls.

Cervical cancer is the second most common cancer in women worldwide (Parkin and Bray, 2006), with the majority of cases and deaths occurring in low-resource countries where organised screening has not been feasible, for example, nearly 20 000 women in Brazil are predicted to develop cervical cancer over the next year (Ferlay *et al*, 2004). Vaccines designed to prevent infections with human papillomavirus (HPV)-16 and -18, responsible for roughly 70% of cases, provide an opportunity for primary prevention. Clinical trials of these vaccines have shown a high degree of efficacy at preventing types 16 and 18 associated infection and precancerous changes in women not previously infected with these types (Harper *et al*, 2006; Ault, 2007; Future II Study Group, 2007; Garland *et al*, 2007; Paavonen *et al*, 2007).

Reductions in cervical cancer mortality by pre-adolescent HPV vaccination would not be observable for many years. Mathematical models that synthesize the best available data while ensuring consistency with epidemiological observations can project outcomes beyond those reported in clinical trials, can provide insights into cost-effectiveness, and can be modified as new information becomes available (Garnett *et al*, 2006; Goldie *et al*, 2007). We recently used an empirically calibrated stochastic model of cervical cancer in a cost-effectiveness analysis of pre-adolescent vaccination of Brazilian girls, with specific attention to strategies that include screening (Goldie *et al*, 2007). Because HPV is sexually transmitted, vaccination of both sexes is being considered in some settings.

To assess the value of including boys in a vaccination programme, a dynamic transmission model is required, which captures not only the direct protective effects on vaccinated individuals, but also the potential indirect effects of reducing HPV transmission to their partners (Edmunds *et al*, 1999; Brisson and Edmunds, 2003; Garnett, 2005). Extending previous work by others (Hughes *et al*, 2002; Taira *et al*, 2004; Elbasha and Galvani, 2005; Barnabas *et al*, 2006; Dasbach *et al*, 2006; French *et al*, 2007), we developed a flexible dynamic model of HPV-16 and -18 sexual transmission between males and females. We linked this model to our stochastic model of cervical carcinogenesis that includes non-vaccine targeted (i.e., non-16, -18) high- and low-risk HPV types to assess the cost-effectiveness of including boys in a pre-adolescent vaccination programme in Brazil.

## MATERIALS AND METHODS

### Dynamic model

**Figure 1:**
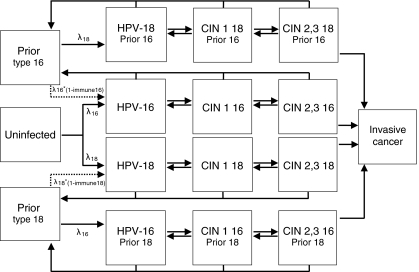
Schematic of dynamic model for females. Females who are uninfected can acquire HPV-16 or -18 infection (at an annual rate of λ_16_ or λ_18_, respectively). Once infected, females can develop precancerous lesions (i.e., CIN 1 and CIN 2,3), and over time may develop invasive cervical cancer. Females who clear their infection or lesion develop a degree of natural immunity to that same HPV type (i.e., immune16 or immune18); future type-specific infections can be acquired at a reduced rate (e.g., λ_16_^*^(1-immune16)). History of prior infection is tracked throughout the analysis. Note: not all health states and transitions are shown. The model for males has a similar structure for HPV-16 and -18 infection only (the schematic and corresponding model equations can be found in the Supplementary information). Once vaccination is introduced, females and males enter a corresponding vaccinated state; vaccine efficacy is modelled as protection against future type-specific infection.

A simplified schematic of the dynamic transmission model is shown in Figure 1. It is an open-cohort, age-structured (ages 0–90 in yearly intervals) compartmental model in which females and males form sexual partnerships over time. Sexually-naive girls and boys enter the susceptible pool upon sexual initiation starting at age 12, and with each partnership, HPV-16 and -18 can be transmitted. Following first HPV infection and clearance, individuals may develop type-specific natural immunity, effectively reducing their susceptibility to future same-type infection. Women with HPV infection can develop cervical intraepithelial neoplasia, grade 1 (CIN 1) or grade 2–3 (CIN 2,3), and those with CIN 2,3 may develop invasive cancer. Males can acquire HPV infection from infected females with or without CIN.

Input parameters for the model were estimated using primary data from longitudinal epidemiological studies and other published literature, and for some inputs, were indirectly estimated using calibration methods. Population and demographic statistics from Brazil were used to inform population size, and birth and death rates (U.S. Census Bureau, 2000; U.N. Population Division, 2004). We adapted a previously published sexual mixing algorithm (Barnabas *et al*, 2006) to reflect data on sexual behaviour in Brazil, such as age of sexual debut and number of sexual partners (U.S.A.I.D., 2006). Briefly, depending on age, members of the male and female cohorts belong to one of four sexual activity groups (none, low, moderate, and high), which govern the number of new sexual partners per year. Each year, type-specific HPV incidence changes according to the number of new partners, HPV prevalence in the opposite gender, and the probability of HPV transmission given a partner has HPV-16 or -18 infection. Details of model structure, input parameters and sexual mixing assumptions are provided in the Supplementary information.

A likelihood-based calibration exercise was used to identify combinations of four uncertain parameter values that produced good model fit to empirical data. These parameters included (1) transmission probability of HPV-16 per infected-susceptible partnership, (2) transmission probability of HPV-18 per infected-susceptible partnership, (3) clearance rate of HPV-16 and -18 infection, and (4) progression rate of CIN 2,3 to invasive cancer. We employed the following approach: more than 100 000 model simulations were run in the absence of vaccination or screening. For each simulation, one value for each of the four parameters was randomly selected from a uniform distribution over pre-specified plausible ranges, creating a unique natural history parameter set. Model outcomes using each parameter set were scored according to their fit with calibration targets established using epidemiological data from studies in Brazil and other South American countries. Good-fitting sets were identified based on a composite goodness-of-fit score. Details of these methods are provided in the Supplementary information.

Using the best-fitting parameter set, we projected reductions in HPV-16 and -18 incidence that would be expected over time with and without HPV vaccination of pre-adolescent girls alone *vs* boys and girls. We assumed vaccination occurred in girls and boys before age 12 and provided lifetime protection against all incident HPV-16 and -18 infections. Coverage rates were varied from 0 to 90% in girls and boys independently. After the epidemic achieved equilibrium post-vaccination, age-specific incidence rates of HPV-16 and -18 were generated for each vaccination scenario. The reductions in HPV incidence projected from the dynamic transmission model were then used as direct inputs to our previously described stochastic model (Goldie *et al*, 2007; Kim *et al*, 2007). The latter model differs from the dynamic transmission model in the following ways: (1) only females are represented; (2) all HPV types (both vaccine-targeted and non-targeted) are included; (3) HPV incidence is a function of age and individual-level characteristics, but does not explicitly change over time in response to sexual activity and population prevalence; (4) it is an individual-based model, which keeps track of each woman's history; and (5) it is stochastic, reflecting variability as well as uncertainty. Like the dynamic transmission model, it is empirically calibrated to epidemiological data (Goldie *et al*, 2007; Kim *et al*, 2007).

### Cost-effectiveness analysis

Lifetime costs, life expectancy, and incremental cost-effectiveness ratios were estimated for including boys in a vaccination programme compared to girls alone. Estimations of costs (e.g., cancer treatment) are documented elsewhere (Goldie *et al*, 2007), but briefly, we included direct medical costs, nonmedical costs, and time costs. Since the price of the vaccine and cost of delivery in Brazil is uncertain, we assumed a composite cost per-vaccinated individual, which was varied from I$25 to 400; for example, for a composite cost of $25 per-vaccinated individual, we assumed three doses of vaccine at $5 each ($15), wastage of $2.25, freight and supplies of $1.31, administration of $1.50, and vaccine support and programmatic costs of $4.94 (Goldie *et al*, 2007). Costs are presented in 2000 international dollars, a currency that provides a means of comparing costs among countries, taking into account differences in purchasing power (World Health Organization, 2007).

Following published guidelines for economic evaluations, we adopted a societal perspective and included all costs and benefits regardless to whom they accrue and discounted future costs and life years by 3% annually (Gold *et al*, 1996). The performance of alternative strategies was measured using the incremental cost-effectiveness ratio, which is defined as the additional cost of a specific strategy, divided by its additional benefit compared with the next-most-expensive strategy. Strategies were excluded from the cost-effectiveness calculations if they were more costly and less effective (i.e., strongly dominated) or less costly and less cost-effective (i.e., weakly dominated) than an alternative strategy. We assessed the model's internal consistency (against data used as inputs), external consistency (against known facts about the disease), projective validity (against data sourced independently from model inputs), and convergent validity (against results from different models) (Weinstein *et al*, 2003).

## RESULTS

### Model calibration and validity

**Figure 2:**
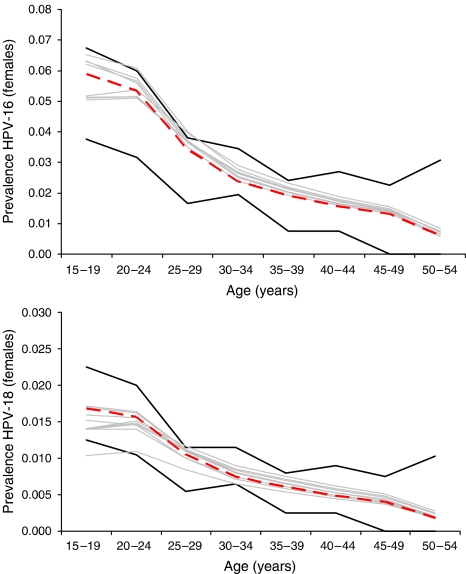
Model output of age-specific prevalence of HPV-16 (upper panel) and HPV-18 (lower panel) among females compared to empirical data. Red dotted line represents model output for the best-fitting set; grey lines represent model output for the next nine best-fitting sets. Black solid lines depict the 95% confidence interval of the empirical data at each age group (Franco *et al*, 1999; Molano *et al*, 2002; Clifford *et al*, 2005, 2006).

**Figure 3:**
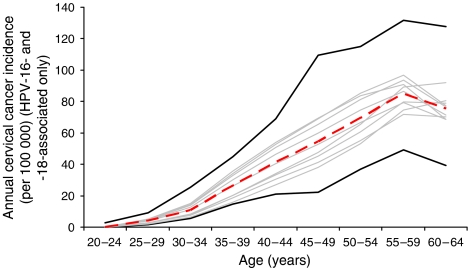
Model output of age-specific incidence of cervical cancer (HPV-16 and-18 associated only) compared to empirical data. Red dotted line represents model output for the best-fitting set; grey lines represent model output for the next nine best-fitting sets. Black solid lines depict the 95% confidence interval of the empirical data at each age group (International Agency for Research on Cancer, 1976; Clifford *et al*, 2003a, 2003b, 2006).

The calibrated values of the four parameters in the best-fitting parameter set, and the mean and range of values for the 10 best-fitting sets, are presented in the Supplementary information. Figure 2 shows examples of model output from the 10 sets with the best goodness-of-fit scores compared with empirical prevalence data of HPV-16 (upper panel) and -18 (lower panel) from two large epidemiological studies in South America (Franco *et al*, 1999; Molano *et al*, 2002; Clifford *et al*, 2005). External consistency of the model was demonstrated by producing outcomes that were within the 95% confidence intervals of independent data, including age-specific cancer incidence rates (HPV-16 and -18 associated only) (International Agency for Research on Cancer, 1976; Clifford *et al*, 2003a, 2003b, 2006) (Figure 3). Additional calibration results and an assessment of projective validity are provided in the Supplementary information.

### Clinical benefits and cost-effectiveness of vaccination

Shown in Table 1 are the reductions in lifetime cancer risk and the cost-effectiveness ratios associated with vaccinating both boys and girls *vs* girls alone at different costs per-vaccinated individual ($25–400) and coverage rates (equal for both genders). In a vaccination programme for pre-adolescent girls alone, benefits were generally proportional to the level of coverage; for example, reduction in overall cancer risk was 14% with 25% coverage, and 63% with 90% coverage. When boys were added to the vaccination programme, cancer reduction was consistently higher than when covering girls alone; however, the magnitude of the incremental benefit of including boys depended on the level of coverage achieved for girls. For example, at 50% coverage of girls, reduction in lifetime risk of cancer increases from 29 to 40% when including equal coverage of boys; in contrast, at 90% coverage, reduction in cancer increases from 63 to 67% when including boys.

At a composite cost of $25 per-vaccinated individual (approximately $5 per dose), vaccinating pre-adolescent girls alone was cost-saving compared to no vaccination, at all coverage levels. When this cost increased to $50 (approximately $12 per dose), vaccination was no longer cost-saving, and the cost-effectiveness ratios varied by level of coverage; while vaccinating girls only was consistently less than $200 per year of life saved (YLS), the ratio for vaccinating both girls and boys increased from $810 per YLS to $18 650 per YLS, as coverage increased. This trend was consistent at higher costs.

Because countries may consider investments to increase vaccine coverage, we explored the tradeoffs associated with increasing coverage in girls *vs* including boys in a vaccination programme (Table 2). At initial coverage levels of 25 or 75% for alone, a strategy of including boys was always more costly and less effective than increasing coverage for girls; for example, when considering investments beyond 25% coverage in girls alone, a strategy of increasing their coverage to 50% provided an 8% greater reduction in cancer risk and was less costly than adding 25% coverage in boys. Even with 75% coverage in girls, increasing their coverage to 90% was more effective and less costly than adding coverage of boys up to 25, 50, or 75%.

Results of other sensitivity analyses, including varying assumptions about type-specific natural immunity, an increase in cancer caused by non-vaccine targeted HPV types, cross-protection extending to other HPV types, and waning vaccine immunity, produced findings similar to those reported in previous analyses (Goldie *et al*, 2007).

## DISCUSSION

The goal of our analysis is to inform discussions about how best to reduce deaths from cervical cancer in Brazil and epidemiologically similar countries. Our results suggest that for a pre-adolescent vaccination programme in which coverage of girls is high, the added value of including boys will be relatively small compared with settings in which coverage of girls is low. While there were increased health benefits for females when boys were included at any coverage level, the marginal impact on cervical cancer incidence diminished as coverage in girls increased, while total costs nearly doubled (assuming same coverage for both genders), resulting in less attractive cost-effectiveness ratios. When we comparatively assessed the impact of increasing coverage in girls to including boys in the vaccination programme, we found that even a modest increase in coverage of girls was less costly, yet more beneficial, than covering an equal percentage of boys. In other words, at any specific coverage level, a decision maker faced with the choice of trying to expand coverage in girls *vs* including boys should always increase coverage in girls first, all else being equal.

The Commission on Macroeconomics and Health has suggested that interventions with ratios below per capita GDP should be considered ‘very cost-effective’ (World Health Organization, 2001). For Brazil, this would imply a threshold of approximately I$ 8600 (U.S. Central Intelligence Agency, 2007). Some would argue that the real-world threshold for a new programme should be the incremental cost-effectiveness ratios of other public health interventions competing for the same resources, such as vaccines that have already been implemented. In this case, the relevant threshold ratio could be as low as $500 per YLS (Jha *et al*, 1998). Adopting this lower threshold would imply that the cost per-vaccinated person would need to approximate or be lower than $50 for pre-adolescent vaccination of girls to be cost-effective. Further, unless this cost is well below $50, coverage in girls is well below 50%, and coverage in girls could not be increased, then adding boys to a vaccination programme may not be cost-effective in Brazil. We acknowledge however, that cost-effectiveness is one of the many factors that influence decision-making and that there may be other considerations, such as equity, community perception, cultural preferences, and political realities that will play a larger role.

As recommended in guidelines for health economic evaluations (Gold *et al*, 1996), we adopted a societal perspective and considered a long time horizon that captures not only the immediate costs of vaccination, but also the future costs averted by preventing cancer in later years. While cost-effectiveness analysis provides information on ‘value for money’, it is not equivalent to providing information on the budget impact to a local decision maker. Although countries need to conduct their own financial analyses, estimating the financial resources required for a vaccination programme over the first few years can provide useful, albeit daunting, qualitative insights. For example, at a cost per-vaccinated girl of $100, vaccinating just 50% of 11-year-old girls is projected to exceed $85 million in just the first year of the programme. Adding boys at the same coverage level would double the cost. Of note, because our analysis presents cost-effectiveness results in international dollars, the relevant financial assessment would convert the tradeable portion of the intervention cost (e.g., vaccine) using the foreign exchange rate, and express all costs in local currency.

Our results associated with vaccinating girls alone were consistent with those obtained in a previous analysis we conducted in Brazil using our stochastic model of girls (Goldie *et al*, 2007). In particular, we found that the cost-effectiveness of HPV vaccination was most influenced by the vaccine price and cost of delivering adolescent vaccination. Although dynamic transmission models of HPV infection are increasingly being developed to explore the population-level impact of an HPV vaccine, to date, there are few that evaluate the cost-effectiveness of vaccinating both boys and girls within vaccination programmes (Taira *et al*, 2004; Dasbach *et al*, 2006; Elbasha *et al*, 2007; Newall *et al*, 2007), and none pertaining to low-resource settings. These studies, conducted in the context of current screening in the United States, have drawn similar conclusions that vaccinating 12-year-old girls is an attractive strategy. However, while Taira *et al* (2004) found that including boys in a vaccination programme costs nearly $450 000 per quality-adjusted life years (QALY) gained, Elbasha *et al* (2007) concluded that vaccinating both boys and girls, with catch-up programmes up to age 24 for both sexes, costs $45 100 per QALY. Although Elbasha *et al* (2007) capture the vaccine benefits of reducing HPV-6 and -11 related sequelae, they do not incorporate the impact of the vaccine on overall cancer incidence associated with all HPV types. Similar to Taira *et al* (2004), we employed two distinct types of models to address this issue and capitalise on the strengths of each. In the dynamic model, we reflected the infectious transmission of HPV-16 and -18 among sexually active males and females to capture herd immunity effects, and in the stochastic model, we translated the benefits of a vaccine targeting two types of HPV to overall cancer incidence associated with all high-risk HPV types.

As with all modelling approaches there are limitations to our analysis that should be noted. First, like others (Hughes *et al*, 2002; Barnabas *et al*, 2006; Burchell *et al*, 2006), we indirectly estimated HPV transmission probabilities per infected-susceptible partnership. Although similar to the calibrated estimates of transmission per coital act by Burchell *et al* (2006), our calibrated values of transmission probability per infected-susceptible partnership for HPV-16 and -18 were lower than those estimated from other studies (Hughes *et al*, 2002; Barnabas *et al*, 2006); however, we modelled a different population with different HPV prevalence and sexual behaviours and allowed other uncertain variables, such as HPV clearance and CIN 2,3 progression to cancer, to simultaneously vary in the fitting process, as well. As better data become available on age- and gender-specific transmission, it will be important to reassess these estimates.

As we await data on vaccine efficacy in boys, we made an assumption that the vaccine was as efficacious in reducing HPV-16 and -18 incidence among boys as it is for girls. Even under such generous assumptions, we found that investing in increased coverage of girls was far more favourable than including boys in a vaccination programme. We did not, however, consider the potential benefits of the quadrivalent HPV vaccine in preventing HPV-6 and -11 associated genital warts, as our analysis was explicitly focused on reducing cervical cancer mortality; any positive externalities that are not included in the analysis would improve the cost-effectiveness of the vaccine in the overall population. We did not include other cancers associated with HPV-16 and -18, as data in Brazil are limited, and also because their natural histories are not well-elucidated to model over decades. Detailed sexual behaviour data are limited. Not only is there severe under-reporting and misreporting of sexual behaviour, but there are also significant time trends that may be occurring in developing countries with respect to age of sexual initiation and number of partners that can impact overall incidence of HPV. To the extent that childhood exposure to HPV (e.g., from sexual abuse or mother-to-child transmission), and in particular HPV-16 and -18, is underestimated, we may be overestimating the protective effects of the vaccine. We did not model bisexual or homosexual partnerships, nor did we include risk factors that may be changing over time, such as smoking (Ho *et al*, 1998; Munoz *et al*, 2006).

We assumed individuals had an equal chance of getting vaccinated, but in reality, uptake may be lower in settings with less access to adolescents, such as rural areas and where children are not in school. It will therefore be important to monitor differential uptake and post-vaccination behaviour. Finally, as we have previously reported, potentially influential uncertainties such as the duration of vaccine efficacy, magnitude of herd immunity, cross protection, interactions between HPV types and natural history of multiple infections all represent data gaps; modelled estimates will improve as better information becomes available.

In light of the range of uncertainties and the unavoidable limitations inherent in modelling methods, we present our findings as exploratory and aim to provide qualitative insight into decisions that countries will be facing in the coming years regarding HPV vaccine implementation. On the basis of the most current epidemiological data, the results of this analysis suggest that the benefits of including boys in an HPV vaccination programme depend on the level of coverage achievable for girls, and in particular, the added benefit on cancer reduction is relatively small provided coverage of girls is greater than 75%. Moreover, even at this coverage level, expanding coverage in girls is more cost-effective than adding boys to the vaccination programme. In a resource-constrained setting such as Brazil, our results support that the first priority in reducing cervical cancer mortality should be to vaccinate pre-adolescent girls.

## Figures and Tables

**Table 1:** Clinical benefits and incremental cost-effectiveness ratios by vaccine coverage and cost per-vaccinated individual^a^

	**Reduction in lifetime cancer risk^b^ %**	**Cost per-vaccinated individual^c^**			
		**$25 ($5 per dose)**	**$50 ($12 per dose)**	**$100 ($27 per dose)**	**$400 ($115 per dose)**
*25*% *coverage*					
Girls only	14	Cost-saving^d^	70	610	3450
Girls and boys	21	110	810	2190	9370
					
*50*% *coverage*					
Girls only	29	Cost-saving^d^	30	540	3210
Girls and boys	40	660	1740	3900	15 120
					
*75*% *coverage*					
Girls only	45	Cost-saving^d^	130	740	3940
Girls and boys	57	2440	2180	4860	18 820
					
*90*% *coverage*					
Girls only	63	Cost-saving^d^	170	810	4180
Girls and boys	67	9110	18 650	37 720	136 910

a Values represent incremental cost-effectiveness ratios (additional cost divided by additional health benefit compared to the next best strategy) expressed as cost per year of life saved (international dollar per YLS). Strategies including girls alone were compared to no vaccination.

b Reduction in lifetime cancer risk for all strategies was calculated against no vaccination.

c Cost per-vaccinated individual includes three doses, wastage, delivery, and programmatic costs, and is expressed in 2000 international dollars.

d Strategies are cost-saving compared to no vaccination because the future costs averted by preventing cancer are greater than the cost of vaccination.

**Table 2:** Tradeoff of increasing vaccine coverage of girls versus including boys^a^

	**Reduction in lifetime cancer risk^b^ %**	**Total cost (I$)**	**Total life expectancy (years)**	**Incremental cost-effectiveness ratios (I$/YLS)**
*If we can achieve beyond 25% vaccine coverage in girls, should we invest in increasing coverage in girls (to 50%) or adding coverage of boys (to 25%)?*				
No vaccination	—	58.47	39.9442	—
Increase girls: girls only (50%)	29	59.87	39.9928	30
Girls only (25%)	14	60.05	39.9671	Dominated^c^
Add boys: girls (25%)+boys (25%)	21	67.33	39.9761	Dominated^c^
*If we can achieve beyond 75% vaccine coverage in girls, should we invest in increasing coverage in girls (to 90%) or including coverage of boys (to 25% or higher)?*				
No vaccination	—	58.47	39.9442	—
Girls only (75%)	45	66.34	40.0051	130
Increase girls: girls only (90%)	63	71.20	40.0213	300
Add boys: girls (75%)+boys (25%)	48	76.23	40.0110	Dominated^c^
Add boys: girls (75%)+boys (50%)	52	86.86	40.0135	Dominated^c^
Add boys: girls (75%)+boys (75%)	57	96.72	40.0191	Dominated^c^

a I$=international dollar; YLS=years of life saved. Analysis assumed cost per-vaccinated individual of $50 (i.e., $12 per dose). Strategies are listed by increasing total cost.

b Reduction in lifetime cancer risk for all strategies was calculated against no vaccination.

c 'Dominated' strategies were more costly and less effective (i.e., strongly dominated) than an alternative strategy of covering girls alone.
