# Supplementary material for: The value of including boys in an HPV vaccination programme: a cost-effectiveness analysis in a low-resource setting
Source: Br J Cancer. 2007 Oct 9;97(9):1322–8. doi: 10.1038/sj.bjc.6604023 (PMC2360471; doi:10.1038/sj.bjc.6604023)
Supplement: Supplementary Information [file 6604023x1.doc]

**The value of including boys in an HPV vaccination program:**

**A cost-effectiveness analysis in a low-resource setting**

Jane J. Kim, Bethany Andres-Beck, Sue J. Goldie

**Technical Appendix**

**Analytic overview of the study.** As shown in the **Figure** below, our dynamic model simulates sexual transmission of HPV-16 and -18 between men and women, by age and sexual activity level. Using population statistics, primary data from longitudinal, epidemiological studies, and cancer registry data from Brazil, we parameterized the baseline model inputs. For four key uncertain parameters of the model, we conducted a calibration exercise to identify combinations of parameter values that produced good model fit to empirical data. Using the best fitting parameter set, we projected the reduction in HPV-16 and -18 incidence that would be expected over time with HPV vaccination policies targeting girls alone versus both boys and girls. These estimates of reduction in HPV-16 and -18 incidence were then used as inputs to our previously described individual-based stochastic model of cervical carcinogenesis (Goldie et al., 2007; Kim et al., 2007). Short and long-term health and economic consequences were assessed for vaccination strategies that focused on girls alone, versus girls and boys.

Parameter Inputs

Calibration

and Validation

Information for

Policy-Making

Population

Stratification

Gender

(Females, Males)

Age

(0-90)

Sexual Activity Level

(None, Low, Mod, High)

×

×

Sex behavior

Demographic

Epidemiological

Interventions

Comparison of model output to independent data

Parameter search

Likelihood-based methods

to fit empirical data

Analysis

Calculate reduction

in HPV-16, -18

incidence with vaccine

Generate HPV-16, -18

incidence

in dynamic model

Apply % reduction to

HPV-16, -18 incidence

in stochastic model

General Structure

of Models

Susceptible

CIN 1

CIN 2,3

Cancer

What is the value of including boys in an HPV vaccination program?

HPV Infection

Movement among health states depend on HPV type, natural immunity, vaccination, screening

**Schematic of dynamic model for females and for males.** Females who are uninfected can acquire HPV 16 or 18 infection (at an annual rate of λ_16_ or λ_18_, respectively). Once infected, females can develop precancerous lesions (i.e., CIN1 and CIN2,3), and over time may develop invasive cervical cancer. Females who clear their infection or lesion develop a degree of natural immunity to that same HPV type (i.e., immune16 or immune18); future type-specific infections can be acquired at a reduced rate (e.g., λ_16_*1-immune16). History of prior infection is tracked throughout the analysis. The model for males has a similar structure for HPV-16 and -18 infection only. Once vaccination is introduced, females and males enter a corresponding vaccinated state; vaccine efficacy is modeled as protection against future type-specific infection.

**Females**

**Uninfected**

**Prior**

**type 18**

**Prior**

**type 16**

**HPV 16**

**HPV 18**

**CIN1 16**

**λ_16_**

**λ_18_**

**HPV 18**

**Prior 16**

**λ_18_**

**HPV 16**

**Prior 18**

**λ_16_**

**CIN2,3 16**

**CIN1 18**

**CIN2,3 18**

**CIN1 16**

**Prior 18**

**CIN2,3 16**

**Prior 18**

**CIN1 18**

**Prior 16**

**CIN2,3 18**

**Prior 16**

**Invasive**

**Cancer**

**λ_18_*(1-immune18)**

**λ_16_*(1-immune16)**

**Males**

**Uninfected**

**Prior**

**type 18**

**Prior**

**type 16**

**HPV 16**

**HPV 18**

**λ_16_**

**λ_18_**

**HPV 18**

**Prior 16**

**λ_18_**

**HPV 16**

**Prior 18**

**λ_16_**

**λ_16_*(1-immune16)**

**λ_18_*(1-immune18)**

**Boundary Conditions**

*Females*

Sw_t_(0,j) = prop_female*$\int_{0}^{\infty} \text{π}(i')*$[$Sw_{t}(i',j)+Iw16_{t}(i',j)+Iw18_{t}(i',j)+L16_{t}(i',j)+L18_{t}(i',j)+H16_{t}(i',j)+H18_{t}(i',j)+CA16_{t}(i',j)+CA18_{t}(i',j)$

$$+Histw16_{t}(i',j)+Histw18_{t}(i',j)+Histw1618_{t}(i',j)+Histw18\_I16_{t}(i',j)+Histw16\_I18_{t}(i',j)$$

$$+Histw18\_L16_{t}(i',j)+Histw16\_L18_{t}(i',j)+Histw18\_H16_{t}(i',j)+Histw16\_H18_{t}(i',j)+Vw_{t}(i',j)]di'$$

*Males*

Sm_t_(0,j) = (1-prop_female)*$\int_{0}^{\infty} \text{π}(i')*$[$Sw_{t}(i',j)+Iw16_{t}(i',j)+Iw18_{t}(i',j)+L16_{t}(i',j)+L18_{t}(i',j)+H16_{t}(i',j)+H18_{t}(i',j)+CA16_{t}(i',j)+CA18_{t}(i',j)$

$$+Histw16_{t}(i',j)+Histw18_{t}(i',j)+Histw1618_{t}(i',j)+Histw18\_I16_{t}(i',j)+Histw16\_I18_{t}(i',j)$$

$$+Histw18\_L16_{t}(i',j)+Histw16\_L18_{t}(i',j)+Histw18\_H16_{t}(i',j)+Histw16\_H18_{t}(i',j)+Vw_{t}(i',j)]di'$$

**State Transition Equations**

*Females*

Sw*_t+1_*(I,j) = Sw*_t_*(I,j) + prop_female*π(i) – [λw16_t_(I,j) + λw18 _t_(I,j) + vacc(i)*efficacy+ µw(i)]*Sw_t_(I,j)

Iw16 *_t+1_*(I,j) = Iw16 *_t_*(I,j) + λw16_t_(I,j)*Sw_t_(I,j) + CIN1regr*(1-CIN1clear)*L16 _t_(I,j) + (1-imm_degree16)* λw16_t_(I,j)*Histw16 _t_(I,j) – [HPVclear + HPVprog + µw(i)]*Iw16 *_t_*(I,j)

Iw18 *_t+1_*(I,j) = Iw18 *_t_*(I,j) + λw18_t_(I,j)*Sw_t_(I,j) + CIN1regr*(1-CIN1clear)*L18 _t_(I,j) + (1-imm_degree18)* λw18_t_(I,j)*Histw18 _t_(I,j) – [HPVclear + HPVprog + µw(i)]*Iw18 *_t_*(I,j)

L16 *_t+1_*(I,j) = L16 *_t_*(I,j) + HPVprog*(propCIN1)*Iw16 *_t_*(I,j) + CIN23regr*(1-CIN23clear)*H16 _t_(I,j) – [CIN1regr + CIN1prog + µw(i)]*L16 _t_(I,j)

L18 *_t+1_*(I,j) = L18 *_t_*(I,j) + HPVprog*(propCIN1)*Iw18 *_t_*(I,j) + CIN23regr*(1-CIN23clear)*H18 _t_(I,j) – [CIN1regr + CIN1prog + µw(i)]*L18 _t_(I,j)

H16 *_t+1_*(I,j) = H16 *_t_*(I,j) + HPVprog(1-propCIN1)*Iw16*_t_*(I,j) + CIN1prog*L16 _t_(I,j) – [CIN23regr + CIN23prog + µw(i)]*H16 _t_(I,j)

H18 *_t+1_*(I,j) = H18 *_t_*(I,j) + HPVprog(1-propCIN1)*Iw18*_t_*(I,j) + CIN1prog*L18 _t_(I,j) – [CIN23regr + CIN23prog + µw(i)]*H18 _t_(I,j)

CA16 *_t+1_*(I,j) = CA16 *_t_*(I,j) + CIN23prog*[H16 _t_(I,j) + Histw18_H16*_t_*(I,j)] – [µw(i) + µCA]*CA16 *_t_*(I,j)

CA18 *_t+1_*(I,j) = CA18 *_t_*(I,j) + CIN23prog*[H18 _t_(I,j) + Histw16_H18*_t_*(I,j)] – [µw(i) + µCA]*CA18 *_t_*(I,j)

Histw16 *_t+1_*(I,j) = Histw16 *_t_*(I,j) + HPVclear*Iw16*_t_*(I,j) + CIN1regr*CIN1clear*L16 _t_(I,j) + CIN23regr*CIN23clear* H16 _t_(I,j) – [(1-imm_degree16)* λw16_t_(I,j) + λw18_t_(I,j) + µw(i)]*Histw16 _t_(I,j)

Histw18 *_t+1_*(I,j) = Histw18 *_t_*(I,j) + HPVclear*Iw18*_t_*(I,j) + CIN1regr*CIN1clear*L18 _t_(I,j) + CIN23regr*CIN23clear* H18 _t_(I,j) – [(1-imm_degree18)* λw18_t_(I,j) + λw16_t_(I,j) + µw(i)]*Histw18 _t_(I,j)

Histw1618 *_t+1_*(I,j) = Histw1618 *_t_*(I,j) + HPVclear*[Histw18_I16 *_t_*(I,j) + Histw16_I18 *_t_*(I,j)] + CIN1regr*CIN1clear*[ Histw18_L16 + Histw16_L18] + CIN23regr*CIN23clear*[ Histw18_H16 + Histw16_H18] – [(1-imm_degree16)* λw16_t_(I,j) + (1-imm_degree18)* λw18_t_(I,j) + µw(i)]*

Histw1618 *_t_*(I,j)

Histw18_I16 *_t+1_*(I,j) = Histw18_I16 *_t_*(I,j) + λw16_t_(I,j)*Histw18 _t_(I,j) + (1-imm_degree16)* λw16_t_(I,j)*Histw1618 *_t_*(I,j) + CIN1regr*(1-CIN1clear)*Histw18_L16 *_t_*(I,j) – [HPVprog + HPVclear + µw(i)]*Histw18_I16 *_t_*(I,j)

Histw16_I18 *_t+1_*(I,j) = Histw16_I18 *_t_*(I,j) + λw18_t_(I,j)*Histw16 _t_(I,j) + (1-imm_degree18)* λw18_t_(I,j)*Histw1618 *_t_*(I,j) + CIN1regr*(1-CIN1clear)*Histw16_L18 *_t_*(I,j) – [HPVprog + HPVclear + µw(i)]*Histw16_I18 *_t_*(I,j)

Histw18_L16 *_t+1_*(I,j) = Histw18_L16 *_t_*(I,j) + HPVprog*propCIN1*Histw18_I16 *_t_*(I,j) + CIN23regr*(1-CIN23clear)* Histw18_H16 *_t_*(I,j) – [CIN1regr + CIN1prog + µw(i)]*Histw18_L16*_t_*(I,j)

Histw16_L18 *_t+1_*(I,j) = Histw16_L18 *_t_*(I,j) + HPVprog*propCIN1*Histw16_I18 *_t_*(I,j) + CIN23regr*(1-CIN23clear)* Histw16_H18 *_t_*(I,j) – [CIN1regr + CIN1prog + µw(i)]*Histw16_L18*_t_*(I,j)

Histw18_H16 *_t+1_*(I,j) = Histw18_H16 *_t_*(I,j) + HPVprog*(1-propCIN1)*Histw18_I16 *_t_*(I,j) + CIN1prog*Histw18_L16*_t_*(I,j) – [CIN23regr + CIN23prog + µw(i)]*Histw18_H16 *_t_*(I,j)

Histw16_H18 *_t+1_*(I,j) = Histw16_H18 *_t_*(I,j) + HPVprog*(1-propCIN1)*Histw16_I18 *_t_*(I,j) + CIN1prog*Histw16_L18*_t_*(I,j) – [CIN23regr + CIN23prog + µw(i)]*Histw16_H18 *_t_*(I,j)

Vw *_t+1_*(I,j) = Vw *_t_*(I,j) + vacc(i)*efficacy*Sw_t_(I,j) - µw(i)*Vw*_t_*(I,j)

*Males*

Sm*_t+1_*(I,j) = Sm*_t_*(I,j) + (1-prop_female)*π(i) – [λm16_t_(I,j) + λm18 _t_(I,j) + vacc(i)*efficacy + µm(i)]*Sm_t_(I,j)

Im16 *_t+1_*(I,j) = Im16 *_t_*(I,j) + λm16_t_(I,j)*Sm_t_(I,j) + (1-imm_degree16)* λm16_t_(I,j)*Histm16 _t_(I,j) – [HPVclear + µm(i)]*Im16 *_t_*(I,j)

Im18 *_t+1_*(I,j) = Im18 *_t_*(I,j) + λm18_t_(I,j)*Sm_t_(I,j) + (1-imm_degree18)* λm18_t_(I,j)*Histm18 _t_(I,j) – [HPVclear + µm(i)]*Im18 *_t_*(I,j)

Histm16 *_t+1_*(I,j) = Histm16 *_t_*(I,j) + HPVclear*Im16*_t_*(I,j) – [(1-imm_degree16)* λm16_t_(I,j) + λm18_t_(I,j) + µm(i)]*Histm16 _t_(I,j)

Histm18 *_t+1_*(I,j) = Histm18 *_t_*(I,j) + HPVclear*Im18*_t_*(I,j) – [(1-imm_degree18)* λm18_t_(I,j) + λm16_t_(I,j) + µm(i)]*Histm18 _t_(I,j)

Histm1618 *_t+1_*(I,j) = Histm1618 *_t_*(I,j) + HPVclear*[Histm18_I16 *_t_*(I,j) + Histm16_I18 *_t_*(I,j)] – [(1-imm_degree16)* λm16_t_(I,j) + (1-imm_degree18)* λm18_t_(I,j) + µm(i)]*Histm1618 *_t_*(I,j)

Histm18_I16 *_t+1_*(I,j) = Histm18_I16 *_t_*(I,j) + λm16_t_(I,j)*Histm18 _t_(I,j) + (1-imm_degree16)* λm16_t_(I,j)*Histm1618 *_t_*(I,j) – [HPVclear + µm(i)]*

Histm18_I16 *_t_*(I,j)

Histm16_I18 *_t+1_*(I,j) = Histm16_I18 *_t_*(I,j) + λm18_t_(I,j)*Histm16 _t_(I,j) + (1-imm_degree18)* λw18_t_(I,j)*Histw1618 *_t_*(I,j) – [HPVclear + µm(i)]*

Histm16_I18 *_t_*(I,j)

Vm *_t+1_*(I,j) = Vm *_t_*(I,j) + vacc(i)*efficacy*Sm_t_(I,j) - µm(i)*Vm*_t_*(I,j)

**Force of infection** (Barnabas et al., 2006)

$$\lambda w16_{t}(i,j)=\sum_{k=1}^{85} \sum_{l=1}^{4} kw(i,j)\cdot\rho w_{t}(i,j,k,l)\cdot\frac{\beta16\cdot\left(\mathrm{Im}16_{t}(k,l)+\text{Histm}18\_\text{I}16_{t}(k,l)\right)}{\text{Nm}_{t}(k,l)}$$

$$\lambda w18_{t}(i,j)=\sum_{k=1}^{85} \sum_{l=1}^{4} kw(i,j)\cdot\rho w_{t}(i,j,k,l)\cdot\frac{\beta18\cdot\left(\mathrm{Im}18_{t}(k,l)+\text{Histm}16\_\text{I}18_{t}(k,l)\right)}{\text{Nm}_{t}(k,l)}$$

$$\lambda m16_{t}(i,j)=\sum_{k=1}^{85} \sum_{l=1}^{4} km(i,j)\cdot\rho m_{t}(i,j,k,l)\cdot\frac{\beta16\cdot\left(\text{Iw}16_{t}(k,l)+\text{L}16_{t}(k,l)+\text{H}16_{t}(k,l)+\text{Histw}18\_\text{I}16_{t}(k,l)+\text{Histw}18\_\text{L}16_{t}(k,l)+\text{Histw}18\_\text{H}16_{t}(k,l)\right)}{\text{Nw}_{t}(k,l)}$$

$$\lambda m18_{t}(i,j)=\sum_{k=1}^{85} \sum_{l=1}^{4} km(i,j)\cdot\rho m_{t}(i,j,k,l)\cdot\frac{\beta18\cdot\left(\text{Iw}18_{t}(k,l)+\text{L}18_{t}(k,l)+\text{H}18_{t}(k,l)+\text{Histw}16\_\text{I}18_{t}(k,l)+\text{Histw}16\_\text{L}18_{t}(k,l)+\text{Histw}16\_\text{H}18_{t}(k,l)\right)}{\text{Nw}_{t}(k,l)}$$

**Sexual Mixing Matrix**

We used a similar sexual mixing algorithm as described by Barnabas et al. (2006):

$$\rho w_{t}(i,j,k,l)=\left[\varepsilon_{1}\cdot\frac{\sum_{l=1}^{4} \text{Nm}_{t}(k,l)\cdot km(k,l)}{\sum_{k=1}^{85} \sum_{l=1}^{4} \text{Nm}_{t}(k,l)\cdot km(k,l)}+(1-\varepsilon_{1})\cdot\delta(i,k)\right]\left[\varepsilon_{2}\cdot\frac{\text{Nm}_{t}(k,l)\cdot km(k,l)}{\sum_{l=1}^{4} \text{Nm}_{t}(k,l)\cdot km(k,l)}+(1-\varepsilon_{2})\cdot\delta(j,l)\right]$$

$$\rho m_{t}(i,j,k,l)=\left[\varepsilon_{1}\cdot\frac{\sum_{l=1}^{4} \text{Nw}_{t}(k,l)\cdot kw(k,l)}{\sum_{k=1}^{85} \sum_{l=1}^{4} \text{Nw}_{t}(k,l)\cdot kw(k,l)}+(1-\varepsilon_{1})\cdot\delta(i,k)\right]\left[\varepsilon_{2}\cdot\frac{\text{Nw}_{t}(k,l)\cdot kw(k,l)}{\sum_{l=1}^{4} \text{Nw}_{t}(k,l)\cdot kw(k,l)}+(1-\varepsilon_{2})\cdot\delta(j,l)\right]$$

Description of model state variables

| *Females* |  |
| --- | --- |
| Sw_t_(I,j) | Susceptible women (age I, sexual activity group j) with no infection and no history of infection at time t |
| Iw16 _t_(I,j) | Women (age I, sexual activity group j) infected with HPV-16 at time t |
| Iw18 _t_(I,j) | Women (age I, sexual activity group j) infected with HPV-18 at time t |
| L16 _t_(I,j) | Women (age I, sexual activity group j) with low-grade precancerous lesion (i.e., CIN 1) associated with HPV-16 at time t |
| L18 _t_(I,j) | Women (age I, sexual activity group j) with low-grade precancerous lesion (i.e., CIN 1) associated with HPV-18 at time t |
| H16 _t_(I,j) | Women (age I, sexual activity group j) with high-grade precancerous lesion (i.e., CIN 2,3) associated with HPV-16 at time t |
| H18 _t_(I,j) | Women (age I, sexual activity group j) with high-grade precancerous lesion (i.e., CIN 2,3) associated with HPV-18 at time t |
| CA16 _t_(I,j) | Women (age I, sexual activity group j) with invasive cancer associated with HPV-16 at time t |
| CA18 _t_(I,j) | Women (age I, sexual activity group j) with invasive cancer associated with HPV-18 at time t |
| Histw16 _t_(I,j) | Women (age I, sexual activity group j) with history of prior HPV-16 infection and clearance at time t |
| Histw18 _t_(I,j) | Women (age I, sexual activity group j) with history of prior HPV-18 infection and clearance at time t |
| Histw1618 _t_(I,j) | Women (age I, sexual activity group j) with history of prior HPV-16 and -18 infections and clearance at time t |
| Histw18_I16 _t_(I,j) | Women (age I, sexual activity group j) with HPV-16 infection who have a history of prior HPV-18 infection at time t |
| Histw16_I18 _t_(I,j) | Women (age I, sexual activity group j) with HPV-18 infection who have a history of prior HPV-16 infection at time t |
| Histw18_L16 _t_(I,j) | Women (age I, sexual activity group j) with CIN1 associated with HPV-16 who have a history of prior HPV-18 infection at time t |
| Histw16_L18 _t_(I,j) | Women (age I, sexual activity group j) with CIN1 associated with HPV-18 who have a history of prior HPV-16 infection at time t |
| Histw18_H16 _t_(I,j) | Women (age I, sexual activity group j) with CIN2,3 associated with HPV-16 who have a history of prior HPV-18 infection at time t |
| Histw16_H18 _t_(I,j) | Women (age I, sexual activity group j) with CIN2,3 associated with HPV-18 who have a history of prior HPV-16 infection at time t |
| Vw _t_(I,j) | Vaccinated women (age I, sexual activity group j) at time t |
| Nw _t_(I,j) | Total number of women (age I, sexual activity group j) at time t |
|  |  |
| *Males* |  |
| Sm_t_(I,j) | Susceptible men (age I, sexual activity group j) with no infection and no history of infection at time t |
| Im16 _t_(I,j) | Men (age I, sexual activity group j) infected with HPV-16 at time t |
| Im18 _t_(I,j) | Men (age I, sexual activity group j) infected with HPV-18 at time t |
| Histm16 _t_(I,j) | Men (age I, sexual activity group j) with history of prior HPV-16 infection and clearance at time t |
| Histm18 _t_(I,j) | Men (age I, sexual activity group j) with history of prior HPV-18 infection and clearance at time t |
| Histm1618 _t_(I,j) | Men (age I, sexual activity group j) with history of prior HPV-16 and -18 infections and clearance at time t |
| Histm18_I16 _t_(I,j) | Men (age I, sexual activity group j) with HPV-16 infection who have a history of prior HPV-18 infection at time t |
| Histm16_I18 _t_(I,j) | Men (age I, sexual activity group j) with HPV-18 infection who have a history of prior HPV-16 infection at time t |
| Vm _t_(I,j) | Vaccinated men (age I, sexual activity group j) at time t |
| Nm _t_(I,j) | Total number of men (age I, sexual activity group j) at time t |

**Description and Values of model parameters ***

| **Variable Name** | **Description** | **Values** | **Source** |
| --- | --- | --- | --- |
| prop_female | proportion of females in the entire population at t=0 | 0.505 | (U.S. Census Bureau, 2000) |
| π(i) | birth rate, by age i | Appendix Table | (U.N. Population Division, 2004) |
| vacc(i) | proportion of the population vaccinated at age i | varied 10-90% | assumed |
| efficacy | degree of vaccine protection against future HPV-16 and -18 infection among those vaccinated | 100% | (Harper et al., 2006; Koutsky & Harper, 2006; Mao et al., 2006) |
| µw(i) | all-cause mortality rate for females in Brazil, by age i | 0.00034 – 0.05817 † | (World Health Organization, 2002) |
| µm(i) | all-cause mortality rate for males in Brazil, by age i | 0.00104 – 0.08288 † | (World Health Organization, 2002) |
| µCA | excess mortality rate for females with invasive cancer | 0.1630 | (National Cancer Institute, 2005) |
| λw16_t_(I,j) | force of HPV-16 infection among women (age I, sexual activity group j) | calculated by model |  |
| λw18_t_(I,j) | force of HPV-18 infection among women (age I, sexual activity group j) | calculated by model |  |
| λm16_t_(I,j) | force of HPV-16 infection among men (age I, sexual activity group j) | calculated by model |  |
| λm18_t_(I,j) | force of HPV-18 infection among men (age I, sexual activity group j) | calculated by model |  |
| kw(I,j) | number of new partners per year for women (age I, sexual activity group j) | Appendix Table | (U.S.A.I.D., 2006) |
| km(I,j) | number of new partners per year for men (age I, sexual activity group j) | Appendix Table | (U.S.A.I.D., 2006) |
| ρw(I,j,k,l) | mixing matrix for women, representing the probability that women of age I and sexual activity group j forms a partnership with men of age k and sexual activity group l | calculated by model | (Barnabas et al., 2006) |
| ρm(I,j,k,l) | mixing matrix for men, representing the probability that men of age I and sexual activity group j forms a partnership with women of age k and sexual activity group l | calculated by model | (Barnabas et al., 2006) |
| β16 | transmission probability of HPV-16 infection per infected-susceptible partnership | 0.310 ‡ | calibrated |
| β18 | transmission probability of HPV-18 infection per infected-susceptible partnership | 0.262 ‡ | calibrated |
| ε_1_ | mixing coefficient by age (0=assortative; 1=random) | 0.3 | assumed |
| ε_2_ | mixing coefficient by sexual activity group (0=assortative; 1=random) | 0.3 | assumed |
| δ(I,k) | identity matrix for age | 1 if i=k; 0 otherwise |  |
| δ(j,l) | identity matrix for sexual activity group | 1 if j=l; 0 otherwise |  |
| HPVprog | probability of progression from HPV to CIN1 or CIN2,3 | 0.0667 § | (Ho et al., 1995 ; Londesborough et al., 1996 ; McCrory et al., 1999; Schlecht et al., 2003) |
| propCIN1 | proportion of women who progress from HPV to CIN1 (versus CIN2,3) | 0.9 | assumed  (Barnabas et al., 2006 ; McCrory et al., 1999) |
| HPVclear | probability of HPV-16 and HPV-18 clearance | 0.1760 || | calibrated  (Franco et al., 1999 ; McCrory et al., 1999) |

**Description of model parameters (cont) ***

| CIN1prog(i) | | probability of progression from CIN1 to CIN2,3, by age i | 0.0167 – 0.6000 † | (Ho et al., 1998 ; Koutsky et al., 1992 ; Nobbenhuis et al., 1999 ; Remmink et al., 1995) |
| --- | --- | --- | --- | --- |
| CIN1regr | | probability of regression from CIN1 | 0.2667 | (McCrory et al., 1999; Schlecht et al., 2003) |
| CIN1clear | | proportion of women who regress from CIN1 and clear their HPV infection | 0.7 | assumed |
| CIN23prog(i) | | probability of progression from CIN2,3 to invasive cancer, by age i | 0.0441 ¶ | calibrated  (National Cancer Institute, 2005) |
| CIN23regr | | probability of regression from CIN2,3 | 0.0583 | (McCrory et al., 1999; Schlecht et al., 2003) |
| CIN23clear | | proportion of women who regress from CIN2,3 and clear their HPV infection | 0.7 | assumed |
| imm_degree16 | | degree of natural immunity following HPV-16 infection and clearance (lifelong) | 0.5047 # | calibrated |
| imm_degree18 | | degree of natural immunity following HPV-18 infection and clearance (lifelong) | 0.5327 # | calibrated |
| * | HPV, human papillomavirus; CIN, cervical intraepithelial neoplasia. Probabilities are annual unless otherwise noted. | | | |
| † | Range represents age-specific probabilities. | | | |
| ‡ | In calibration process, baseline probability was allowed to vary from 0.1 to 1.0. | | | |
| § | A proportion of females (10%) with HPV who progress to CIN 1 transition directly to CIN 2,3. | | | |
| || | In calibration process, a baseline probability of 0.2667 was allowed to vary by factor of 0-2. | | | |
| ¶ | In calibration process, a baseline probability of 0.0130 was allowed to vary by factor of 1-6. | | | |
| # | Natural immunity represents the degree of protection individuals face against future type-specific infection after first infection and clearance; the values for type-specific natural immunity were obtained from a separate calibration exercise using the stochastic model. | | | |

Brazil Demographic Data

| **Age** | **Population Size (2000)**  (U.S. Census Bureau, 2000)  *Males* | **Population Size (2000)**  (U.S. Census Bureau, 2000)  *Females* | **Birth Rate (2004)**  (U.N. Population Division, 2004)  (annual, per woman) |
| --- | --- | --- | --- |
| 0-4 | 8464596 | 8131962 | --- |
| 5-9 | 8435011 | 8115714 | --- |
| 10-14 | 8896482 | 8580957 | --- |
| 15-19 | 8956122 | 8690058 | 0.0162 |
| 20-24 | 8588098 | 8414374 | 0.0938 |
| 25-29 | 7896446 | 7804041 | 0.1750 |
| 30-34 | 7293533 | 7274230 | 0.1236 |
| 35-39 | 6440717 | 6537662 | 0.0485 |
| 40-44 | 5402122 | 5576433 | 0.0103 |
| 45-49 | 4452219 | 4688854 | 0.0005 |
| 50-54 | 3524425 | 3813385 | --- |
| 55-59 | 2689316 | 3022689 | --- |
| 60-64 | 2106410 | 2490390 | --- |
| 65-69 | 1566991 | 1977744 | --- |
| 70-74 | 1086785 | 1524820 | --- |
| 75-79 | 660617 | 1037736 | --- |
| 80+ | 472243 | 939589 | --- |

**Proportion of Females and Males in Each Sexual Activity Group By Age**

|  | **Sexual Activity Group (Number of New Partners Per Year)**  (U.S.A.I.D., 2006) | | | |
| --- | --- | --- | --- | --- |
| **Age (years)** | **None (0)** | **Low (1-2)** | **Moderate (3-4)** | **High (5+)** |
| ***Females*** |  |  |  |  |
| 12-19 | 0.672 | 0.273 | 0.041 | 0.014 |
| 20-24 | 0.575 | 0.319 | 0.094 | 0.012 |
| 25-29 | 0.753 | 0.201 | 0.035 | 0.012 |
| 30-34 | 0.790 | 0.171 | 0.030 | 0.010 |
| 35-39 | 0.801 | 0.163 | 0.027 | 0.009 |
| 40-44 | 0.815 | 0.152 | 0.025 | 0.008 |
| 45-49 | 0.938 | 0.031 | 0.023 | 0.008 |
| ***Males*** |  |  |  |  |
| 12-19 | 0.508 | 0.369 | 0.081 | 0.043 |
| 20-24 | 0.667 | 0.167 | 0.125 | 0.042 |
| 25-29 | 0.704 | 0.148 | 0.111 | 0.037 |
| 30-34 | 0.723 | 0.139 | 0.104 | 0.035 |
| 35-39 | 0.727 | 0.137 | 0.102 | 0.034 |
| 40-44 | 0.738 | 0.131 | 0.098 | 0.033 |
| 45-49 | 0.745 | 0.128 | 0.096 | 0.032 |

**Dynamic Model Calibration Approach**

Four uncertain natural history parameters were selected for calibration: (1) transmission probability of HPV-16 per infected-susceptible partnership, (2) transmission probability of HPV-18 per infected-susceptible partnership, (3) clearance rate of HPV-16 and -18 infection, and (4) progression rate of CIN 2,3 to invasive cancer. For the transmission probabilities of HPV-16 and -18, we searched across a range of prior probabilities from 0.10 to 1.0; for HPV clearance and CIN 2,3 progression, we identified a plausible range of values using data from the published literature (Franco et al., 1999; McCrory et al., 1999; National Cancer Institute, 2005).

More than 100,000 model simulations were run in the absence of any vaccination or screening intervention. For each simulation, one value for each of the four parameters was randomly selected from a uniform distribution over the identified plausible ranges, creating a unique natural history parameter set. Model outcomes using each parameter set were scored according to their simultaneous fit with calibration target data that were based on epidemiological data from studies in Brazil and other South American countries (see **Table** below).

We specified likelihood functions for all calibration targets, assuming that each followed an independent normal distribution. For each of the 100,000+ parameter sets, we computed a composite goodness-of-fit score by summing over the individual log likelihood measures of all targets. Based on the goodness-of-fit score, we identified the best fitting set to proceed with the analysis.

**Dynamic Model Calibration Target Data**

| **Calibration Target** | **Mean (SD)** |
| --- | --- |
| **Prevalence of HPV-16 infection among women**  (Clifford et al., 2006; Clifford et al., 2005a; Franco et al., 1999; Molano et al., 2002) |  |
| - 15-19 years | 0.0525 (0.0077) |
| - 20-24 years | 0.0458 (0.0073) |
| - 25-29 years | 0.0255 (0.0046) |
| - 30-34 years | 0.0270 (0.0038) |
| - 35-39 years | 0.0158 (0.0042) |
| - 40-44 years | 0.0173 (0.0050) |
| - 45-49 years | 0.0113 (0.0057) |
| - 50-54 years | 0.0154 (0.0078) |
| - 55-59 years | 0.0221 (0.0109) |
| - 60-64 years | 0.0510 (0.0222) |
| - 65-69 years | 0.0353 (0.0180) |
| **Prevalence of HPV-18 infection among women**  (Clifford et al., 2006; Clifford et al., 2005a; Franco et al., 1999; Molano et al., 2002) |  |
| - 15-19 years | 0.0175 (0.0026) |
| - 20-24 years | 0.0153 (0.0024) |
| - 25-29 years | 0.0085 (0.0015) |
| - 30-34 years | 0.0090 (0.0013) |
| - 35-39 years | 0.0053 (0.0014) |
| - 40-44 years | 0.0058 (0.0017) |
| - 45-49 years | 0.0038 (0.0019) |
| - 50-54 years | 0.0051 (0.0026) |
| - 55-59 years | 0.0074 (0.0036) |
| - 60-64 years | 0.0170 (0.0074) |
| - 65-69 years | 0.0118 (0.0060) |
| **Prevalence of CIN 1 (HPV-16 and -18)**  (Clifford et al., 2005b; Lawson et al., 1998; Sadeghi et al., 1988) |  |
| - 15-19 years | 0.0163 (0.0055) |
| - 20-24 years | 0.0168 (0.0056) |
| - 25-29 years | 0.0147 (0.0050) |
| - 30-34 years | 0.0153 (0.0054) |
| - 35-39 years | 0.0150 (0.0064) |
| - 40-44 years | 0.0134 (0.0056) |
| - 45-49 years | 0.0160 (0.0082) |
| - 50-54 years | 0.0221 (0.0113) |
| - 55-59 years | 0.0158 (0.0081) |
| - 60-64 years | 0.0234 (0.0119) |
| - 65-69 years | 0.0153 (0.0078) |

**Dynamic Model Calibration Target Data (cont)**

| **Calibration Target** | | | **Mean (SD)** |
| --- | --- | --- | --- |
| **Prevalence of CIN 2,3 (HPV-16 and -18)** †  (Clifford et al., 2003a; Lawson et al., 1998; Sadeghi et al., 1988) | | |  |
| - 25-29 years | | | 0.0055 (0.0028) |
| - 30-34 years | | | 0.0059 (0.0030) |
| - 35-39 years | | | 0.0064 (0.0033) |
| **Incidence rate of invasive cancer (HPV-16 and -18)** (per 100,000)  (Clifford et al., 2006; Clifford et al., 2003a; Clifford et al., 2003b; International Agency for Research on Cancer, 1976) | | | |
| - 20-24 years | | 1.4 (0.7) | |
| - 25-29 years | | 5.2 (1.9) | |
| - 30-34 years | | 15.5 (5.1) | |
| - 35-39 years | | 29.7 (7.7) | |
| - 40-44 years | | 44.9 (12.2) | |
| - 45-49 years | | 65.8 (22.2) | |
| - 50-54 years | | 75.9 (19.9) | |
| - 55-59 years | | 90.5 (21.0) | |
| - 60-64 years | | 83.5 (22.6) | |
| - 65-69 years | | 69.2 (17.1) | |
| - 70-74 years | | 90.5 (32.1) | |
| - 75-79 years | | 69.1 (27.6) | |
| **Prevalence of HPV-16 and -18 infection among me**  (Franceschi et al., 2002) | |  | |
| - 25-29 years | | 0.1000 (0.0255) | |
| - 30-34 years | | 0.0500 (0.0255) | |
| - 35-39 years | | 0.0250 (0.0128) | |
| - 40-44 years | | 0.0550 (0.0179) | |
| - 45-49 years | | 0.0450 (0.0179) | |
| - 50-54 years | | 0.0300 (0.0153) | |
| - 55-59 years | | 0.0375 (0.0140) | |
| - 60-64 years | | 0.0275 (0.0140) | |
| * | SD, standard deviation; HPV, human papillomavirus; CIN, cervical intraepithelial neoplasia. All target data were assumed to follow normal distributions. | | |
| † | For prevalence of CIN 2,3, small sample size in the data limited the number of age-specific targets. | | |

**Calibrated Parameter Values for Best-Fitting Sets***

| **Variable** | | **Baseline Probability** | **Parameter Search Range** | **Best-Fitting Parameter Set** | **10 Best-Fitting Parameter Sets**  mean (range) |  |
| --- | --- | --- | --- | --- | --- | --- |
| Transmission probability per infected-susceptible partnership | | | | | |  |
| - HPV-16 | | -- | 0.1 – 1.0 | 0.310 | 0.392  (0.299-0.493) |  |
| - HPV-18 | | -- | 0.1 – 1.0 | 0.262 | 0.326  (0.248-0.412) |  |
| CIN 2,3 to invasive cancer (HPV-16 and -18)  (National Cancer Institute, 2005) | | 0.0130 | 1 – 6 † | 3.392 | 2.479  (1.413-3.856) |  |
| HPV clearance  (HPV-16 and -18)  (Franco et al., 1999; McCrory et al., 1999) | | 0.2667 | 0 – 2 † | 0.660 | 0.877  (0.587-1.178) |  |
| * | HPV, human papillomavirus; CIN, cervical intraepithelial neoplasia. Baseline probabilities are annual probabilities. | | | | | |
| † | Values represent factors that were multiplied to the baseline probability. | | | | | |

**Additional Calibration Output**

In addition to the calibration output included in the main paper, the model achieved consistent fit with HPV-16 and -18 prevalence by age in males, using the best-fitting parameter set. Red line represents model output for best-fitting set; gray lines represent model output for top nine best-fitting sets. Black dotted lines depict the 95% confidence interval of the empirical data at each age group (Franceschi et al., 2002).

Prevalence HPV-16 and -18 (Males)

Age (years)

**Projective Validity**

Although demonstrations of consistency with calibration data are important for model parameterization, we also evaluated the projective validity of the model by comparing model predictions of reductions in cervical cancer mortality associated with Pap smear screening to those observed in empirical studies (see **Table** below). With no intervention, the model predicted mortality rates similar to those reported for Brazil by IARC (Ferlay et al., 2004). When we superimposed screening interventions, we found that model-predicted reductions in mortality rates were consistent with those observed in real populations (Raffle et al., 2003; Zeferino et al., 2006).

Projective Validity

| **Outcome** | **Data** | **Model** | **Source** |
| --- | --- | --- | --- |
| Cervical cancer rate per 100,000 (crude)  No intervention | 9.4 | 8.9 | (Ferlay et al., 2004) |
| Cervical cancer mortality reduction in Sao Paulo (%)  Pap smear screening every 3 years, 40% coverage | 20.6 – 37.5 | 35.4 | (Zeferino et al., 2006) |
| Cervical cancer mortality reduction in UK (%)  Pap smear screening every 5 years, 100% coverage | 40.7 – 49.6 | 43.9 | (Raffle et al., 2003) |

**Linkage of dynamic model to stochastic model**

The dynamic model was run under various scenarios of vaccination (i.e., no vaccination, coverage levels varied from 10% to 90% for girls and boys) and age-specific incidence curves for HPV-16 and -18 are generated each year using the force of infection (λ) equations above.

After the epidemic achieved equilibrium post-vaccination, we calculated the reduction in HPV-16 and -18 incidence among women under the various coverage scenarios, compared to no vaccination. Reductions in age-specific HPV-16 and -18 incidence calculated from the dynamic model are then applied directly to the input age-specific HPV-16 and -18 incidence curves of the stochastic model (see simplified model schematic of stochastic model and linkage in the **Figure** below).

Details of the stochastic model structure, assumptions, and calibration are documented elsewhere (Kim et al., 2007). Briefly, the stochastic model was calibrated using a similar likelihood-based approach and has a similar structure to the dynamic model, but offers the following key features: (1) only females are represented; (2) other HPV types are included, categorized as other high-risk types and low-risk types; (3) HPV incidence is a function of age and individual-level characteristics, but does not explicitly change over time in response to sexual activity and population prevalence; (4) it is an individual-based model, which reflects detailed heterogeneities among females, such as history of screening and/or treatment, and keeps track of individual-level expenditures; (5) it is stochastic, thereby able to capture variability as well as uncertainty; (6) it is empirically calibrated to multiple epidemiological data associated with all HPV types; and (7) analyses can be run with a single birth cohort or multiple birth cohorts (Goldie et al., 2007; Kim et al., 2007). Because we used two distinct models to estimate the long-term reduction in cervical cancer incidence, we carefully examined the consistency of parameter values and assumptions between the two models. The most important of these included type-specific immunity following clearance of first infection; we estimated these values in a separate calibration exercise using our stochastic model (Kim et al., 2007), and then held these values constant in the dynamic model.

Equations for the dynamic model were written and solved in Matlab; equations for the stochastic model were written and solved in C++.

Cancer^3^

Reduction in HPV Incidence

(from Dynamic Model)

**Progression^2^**

Normal

CIN 2,3

CIN 1

HPV Infected

**Infection^1^**

*Clearance*

*Regression*

Death^4^

^1^ Incidence of infection depends on age, HPV type, prior infection, and type-specific immunity.

^2^ Progression of HPV infection and CIN 1 depends on age and HPV type.

^3^ Cancer states stratified by stage (local, regional, distant) and detection status (undetected, symptom-detected, screen-detected).

^4^ Death can occur from all-cause mortality from every health state and excess cancer-specific mortality from cancer states.

**Model Cost Parameters***

| **Costs (2000 international dollars)** †† | |  |
| --- | --- | --- |
| Vaccine | | 25 - 400 |
| Local invasive cancer  (Arredondo et al., 1995; Pinotti et al., 2000; World Health Organization, 2007) | | 5,145 |
| Regional invasive cancer  (Arredondo et al., 1995; Pinotti et al., 2000; World Health Organization, 2007) | | 4,318 |
| Distant invasive cancer  (Arredondo et al., 1995; Pinotti et al., 2000; World Health Organization, 2007) | | 4,318 |
| * | Costs are presented in 2000 international dollars, a currency that provides a means of translating and comparing costs among countries, taking into account differences in purchasing power (World Health Organization, 2007). | |

**Age-specific HPV-16 incidence in females by coverage, 50 years post-vaccination**

HPV-16 Incidence (per 100,000)

Age (years)

**Age-specific HPV-18 incidence in females by coverage, 50 years post-vaccination**

HPV-18 Incidence (per 100,000)

Age (years)

**Reduction in lifetime risk of overall cervical cancer (associated with all high-risk types) at varying levels of vaccination coverage of girls and boys**

0% Coverage of Boys

10% Coverage of Boys

25% Coverage of Boys

50% Coverage of Boys

75% Coverage of Boys

90% Coverage of Boys

Reduction in Lifetime Risk of Cervical Cancer

(All High-Risk HPV types)

0%

10%

20%

30%

40%

50%

60%

70%

80%

Coverage of Girls (%)

10

25

50

75

90

**Comparison of cancer reduction with and without inclusion of herd immunity effects**

One of the advantages of using a dynamic model to evaluate HPV vaccination is the ability to capture the herd immunity effects of the vaccination program where the benefits of vaccination are experienced not only by those who directly received the vaccine, but also by their partners through reduced transmission. In the case of HPV vaccination, herd immunity effects can result from vaccinating girls and boys (by reducing transmission directly to their partners), as well as from vaccinating girls only (by reducing transmission to their male partners, who then reduce transmission to other female partners). By comparing model output from the stochastic model of females only, which does not reflect indirect effects of vaccination, to those from the dynamic model, we were able to estimate the herd immunity effects of vaccinating girls alone in the population (see **Figure** below). We found that the level of herd immunity, expressed as the incremental reduction in lifetime cancer risk (HPV-16 and -18 associated only) comparing the dynamic and stochastic models, varied by coverage achieved among girls; herd immunity was low when coverage levels were either very low (i.e., 10%) or very high (i.e., 90%), and was higher when coverage levels were moderate (i.e., 50% and 75%).

Reduction in Lifetime Risk of Cervical Cancer

(HPV-16 and -18 associated only)

0%

10%

20%

30%

40%

50%

60%

70%

80%

90%

100%

10

50

75

90

Dynamic model

Stochastic model

Coverage of Girls (%)

2.7%

9.5%

9.3%

5.2%

**References**

Arredondo, A., Lockett, L.Y. & de Icaza, E. (1995). Cost of diseases in Brazil: breast cancer, enteritis, cardiac valve disease and bronchopneumonia. *Rev Saude Publica*, **29,** 349-54.

Barnabas, R.V., Laukkanen, P., Koskela, P., Kontula, O., Lehtinen, M. & Garnett, G.P. (2006). Epidemiology of HPV 16 and cervical cancer in Finland and the potential impact of vaccination: mathematical modelling analyses. *PLoS Med*, **3,** e138.

Clifford, G., Franceschi, S., Diaz, M., Munoz, N. & Villa, L.L. (2006). Chapter 3: HPV type-distribution in women with and without cervical neoplastic diseases. *Vaccine*, **24 Suppl 3,** S26-34.

Clifford, G.M., Gallus, S., Herrero, R., Munoz, N., Snijders, P.J., Vaccarella, S., Anh, P.T., Ferreccio, C., Hieu, N.T., Matos, E., Molano, M., Rajkumar, R., Ronco, G., de Sanjose, S., Shin, H.R., Sukvirach, S., Thomas, J.O., Tunsakul, S., Meijer, C.J. & Franceschi, S. (2005a). Worldwide distribution of human papillomavirus types in cytologically normal women in the International Agency for Research on Cancer HPV prevalence surveys: a pooled analysis. *Lancet*, **366,** 991-8.

Clifford, G.M., Rana, R.K., Franceschi, S., Smith, J.S., Gough, G. & Pimenta, J.M. (2005b). Human papillomavirus genotype distribution in low-grade cervical lesions: comparison by geographic region and with cervical cancer. *Cancer Epidemiol Biomarkers Prev*, **14,** 1157-64.

Clifford, G.M., Smith, J.S., Aguado, T. & Franceschi, S. (2003a). Comparison of HPV type distribution in high-grade cervical lesions and cervical cancer: a meta-analysis. *Br J Cancer*, **89,** 101-5.

Clifford, G.M., Smith, J.S., Plummer, M., Munoz, N. & Franceschi, S. (2003b). Human papillomavirus types in invasive cervical cancer worldwide: a meta-analysis. *Br J Cancer*, **88,** 63-73.

Franceschi, S., Castellsague, X., DalMaso, L., Smith, J.S., Plummer, M., Ngelangel, C., Chichareon, S., Eluf-Neto, J., Shah, K.V., Snijders, P.J.F., Meijer, C.J.L.M., Bosch, F.X. & Munoz, N. (2002). Prevalence and determinants of human papillomavirus genital infection in men. *Br J Cancer*, **86,** 705-711.

Franco, E.L., Villa, L.L., Sobrinho, J.P., Prado, J.M., Rousseau, M.C., Desy, M. & Rohan, T.E. (1999). Epidemiology of acquisition and clearance of cervical human papillomavirus infection in women from a high-risk area for cervical cancer. *J Infect Dis*, **180,** 1415-23.

Harper, D.M., Franco, E.L., Wheeler, C.M., Moscicki, A.B., Romanowski, B., Roteli-Martins, C.M., Jenkins, D., Schuind, A., Costa Clemens, S.A. & Dubin, G. (2006). Sustained efficacy up to 4.5 years of a bivalent L1 virus-like particle vaccine against human papillomavirus types 16 and 18: follow-up from a randomised control trial. *Lancet*, **367,** 1247-55.

Ho, G.Y., Burk, R.D., Klein, S., Kadish, A.S., Chang, C.J., Palan, P., Basu, J., Tachezy, R., Lewis, R. & Romney, S. (1995). Persistent genital human papillomavirus infection as a risk factor for persistent cervical dysplasia. *J Natl Cancer Inst*, **87,** 1365-71.

Ho, G.Y., Kadish, A.S., Burk, R.D., Basu, J., Palan, P.R., Mikhail, M. & Romney, S.L. (1998). HPV 16 and cigarette smoking as risk factors for high-grade cervical intra-epithelial neoplasia. *Int J Cancer*, **78,** 281-5.

International Agency for Research on Cancer. (1976). *Cancer Incidence in Five Continents, vol. 3*. Vol. 3. IARC Scientific Publications No. 15. IARCPress: Lyon.

Kim, J.J., Kuntz, K.M., Stout, N.K., Mahmud, S., Villa, L.L., Franco, E.L. & Goldie, S.J. (2007). Multiparameter calibration of a natural history model of cervical cancer. *Am J Epidemiol*, **166,** 137-50.

Koutsky, L.A. & Harper, D.M. (2006). Chapter 13: Current findings from prophylactic HPV vaccine trials. *Vaccine*, **24 Suppl 3,** S114-21.

Koutsky, L.A., Holmes, K.K., Critchlow, C.W., Stevens, C.E., Paavonen, J., Beckmann, A.M., DeRouen, T.A., Galloway, D.A., Vernon, D. & Kiviat, N.B. (1992). A cohort study of the risk of cervical intraepithelial neoplasia grade 2 or 3 in relation to papillomavirus infection. *N Engl J Med*, **327,** 1272-8.

Lawson, H.W., Lee, N.C., Thames, S.F., Henson, R. & Miller, D.S. (1998). Cervical cancer screening among low-income women: results of a national screening program, 1991-1995. *Obstet Gynecol*, **92,** 745-52.

Londesborough, P., Ho, L., Terry, G., Cuzick, J., Wheeler, C. & Singer, A. (1996). Human papillomavirus genotype as a predictor of persistence and development of high-grade lesions in women with minor cervical abnormalities. *Int J Cancer*, **69,** 364-8.

Mao, C., Koutsky, L.A., Ault, K.A., Wheeler, C.M., Brown, D.R., Wiley, D.J., Alvarez, F.B., Bautista, O.M., Jansen, K.U. & Barr, E. (2006). Efficacy of human papillomavirus-16 vaccine to prevent cervical intraepithelial neoplasia: a randomized controlled trial. *Obstet Gynecol*, **107,** 18-27.

McCrory, D., Mather, D., Bastian, L., Datta, S., Hasselblad, V., Hickey, J., Myers, E. & Nanda, K. (1999). Evaluation of Cervical Cytology. Evidence Report/Technology Assessment No. 5. AHCPR: Rockville.

Molano, M., Posso, H., Weiderpass, E., van den Brule, A.J., Ronderos, M., Franceschi, S., Meijer, C.J., Arslan, A. & Munoz, N. (2002). Prevalence and determinants of HPV infection among Colombian women with normal cytology. *Br J Cancer*, **87,** 324-33.

National Cancer Institute. (2005). Surveillance, Epidemiology, End Results (SEER) Cancer Statistics Review, 1975-2001, Vol. 2005.

Nobbenhuis, M.A., Walboomers, J.M., Helmerhorst, T.J., Rozendaal, L., Remmink, A.J., Risse, E.K., van der Linden, H.C., Voorhorst, F.J., Kenemans, P. & Meijer, C.J. (1999). Relation of human papillomavirus status to cervical lesions and consequences for cervical-cancer screening: a prospective study. *Lancet*, **354,** 20-5.

Pinotti, J.A., Tojal, M.L., Nisida, A.C. & Pinotti, M. (2000). Integrated approach to women's health. *Int J Gynaecol Obstet*, **70,** 191-8.

Raffle, A.E., Alden, B., Quinn, M., Babb, P.J. & Brett, M.T. (2003). Outcomes of screening to prevent cancer: analysis of cumulative incidence of cervical abnormality and modelling of cases and deaths prevented. *BMJ*, **326,** 901.

Remmink, A.J., Walboomers, J.M., Helmerhorst, T.J., Voorhorst, F.J., Rozendaal, L., Risse, E.K., Meijer, C.J. & Kenemans, P. (1995). The presence of persistent high-risk HPV genotypes in dysplastic cervical lesions is associated with progressive disease: natural history up to 36 months. *Int J Cancer*, **61,** 306-11.

Sadeghi, S.B., Sadeghi, A. & Robboy, S.J. (1988). Prevalence of dysplasia and cancer of the cervix in a nationwide, planned parenthood population. *Cancer*, **61,** 2359-61.

Schlecht, N.F., Platt, R.W., Duarte-Franco, E., Costa, M.C., Sobrinho, J.P., Prado, J.C., Ferenczy, A., Rohan, T.E., Villa, L.L. & Franco, E.L. (2003). Human papillomavirus infection and time to progression and regression of cervical intraepithelial neoplasia. *J Natl Cancer Inst*, **95,** 1336-43.

U.N. Population Division. (2004). World Population Prospects: The 2004 Revision Population Database. http://esa.un.org/unpp/: Last accessed on January 19, 2007.

U.S. Census Bureau. (2000). Population Estimates Program, Population Division: Washington, D.C. http://www.census.gov/ipc/www/idbnew.html: Last accessed on January 19, 2007.

U.S.A.I.D. (2006). Demographic and Health Surveys. http://www.measuredhs.com/: Last accessed on January 19, 2007.

World Health Organization. (2002). *The World Health Report 2002: Reducing Risks, Promoting Health Life.* WHO: Geneva.

Zeferino, L.C., Pinotti, J.A., Jorge, J.P., Westin, M.C., Tambascia, J.K. & Montemor, E.B. (2006). Organization of cervical cancer screening in Campinas and surrounding region, Sao Paulo State, Brazil. *Cad Saude Publica*, **22,** 1909-14.
